# Detection of Lung Nodules in Micro-CT Imaging Using Deep Learning

**DOI:** 10.3390/tomography7030032

**Published:** 2021-08-07

**Authors:** Matthew D. Holbrook, Darin P. Clark, Rutulkumar Patel, Yi Qi, Alex M. Bassil, Yvonne M. Mowery, Cristian T. Badea

**Affiliations:** 1 Quantitative Imaging and Analysis Lab, Department of Radiology, Duke University Medical Center, Durham, NC 27710, USA; matthew.holbrook@duke.edu (M.D.H.); darin.clark@duke.edu (D.P.C.); yi.qi@duke.edu (Y.Q.); 2 Department of Radiation Oncology, Duke University Medical Center, Durham, NC 27710, USA; rutulkumar.patel@duke.edu (R.P.); alex.bassil@duke.edu (A.M.B.); yvonne.mowery@duke.edu (Y.M.M.); 3 Department of Head and Neck Surgery & Communication Sciences, Duke University Medical Center, Durham, NC 27710, USA

**Keywords:** preclinical imaging, machine learning (ML), X-ray CT (CT)

## Abstract

We are developing imaging methods for a co-clinical trial investigating synergy between immunotherapy and radiotherapy. We perform longitudinal micro-computed tomography (micro-CT) of mice to detect lung metastasis after treatment. This work explores deep learning (DL) as a fast approach for automated lung nodule detection. We used data from control mice both with and without primary lung tumors. To augment the number of training sets, we have simulated data using real augmented tumors inserted into micro-CT scans. We employed a convolutional neural network (CNN), trained with four competing types of training data: (1) simulated only, (2) real only, (3) simulated and real, and (4) pretraining on simulated followed with real data. We evaluated our model performance using precision and recall curves, as well as receiver operating curves (ROC) and their area under the curve (AUC). The AUC appears to be almost identical (0.76–0.77) for all four cases. However, the combination of real and synthetic data was shown to improve precision by 8%. Smaller tumors have lower rates of detection than larger ones, with networks trained on real data showing better performance. Our work suggests that DL is a promising approach for fast and relatively accurate detection of lung tumors in mice.

## 1. Introduction

Small animal imaging has become essential in evaluating new cancer therapies as they are translated from the preclinical to clinical domain. Longitudinal imaging can be used to non-invasively screen for tumors, monitor growth, and assess changes with treatment. Co-clinical trials are a growing application of small animal imaging where animal results are used to shape ongoing clinical trials [1,2]. In a co-clinical trial, an animal and a clinical arm are run in parallel to study the efficacy of a novel drug or therapy. Ideally, the clinical arm will lag behind the preclinical arm, allowing for real-time integration of animal data in the clinical trial which leads to improved study design and better outcomes for the clinical trial [3].

We are developing quantitative imaging methods for use in the preclinical arm of a co-clinical trial investigating synergy between immunotherapy and radiotherapy for the treatment of soft tissue sarcoma [4,5]. A critical metric of therapeutic response in patients is metastasis-free survival determined through serial imaging of the lungs, the most common site for metastasis for this type of soft tissue sarcoma. Micro-CT has been successfully used to detect lung tumors and evaluate lung tumor burden in many mouse models [6,7]. To mimic the clinical arm of the study and patient standard of care, we perform periodic micro-CT scans of free-breathing mice to detect lung metastasis after treatment. Micro-CT screening allows for longitudinal identification and monitoring of lung abnormalities. This includes monitoring the development of tumor nodules and their response to treatment. We carefully optimized micro-CT for lung nodule imaging using prospective gating, and we performed image quality evaluations and tested repeatability and sensitivity of detection [5]. To date, lung nodules have been detected and measured using manual and semi-automatic procedures. Similar to [8,9,10], we used laborious inspection and manual segmentations of lung tumors, which are slow and may introduce human bias.

Deep learning has emerged as a promising approach for automatic lung nodule detection and classification in human data [11]. Many studies have been carried out using DL algorithms applied to human CT data [12,13,14,15]. These have shown the abilities of DL methods to detect lung nodules at a high level of accuracy, sensitivity, and specificity when applied to annotated archives of clinical scans. As reported in [16], the sensitivity to detect pulmonary nodules using convolutional neural networks (CNNs) ranges from 83.19% to 94%. These CNN approaches are aided by the availability of several publicly available CT datasets with lung nodules available for training and validation. These include the LIDC/IDRI dataset [17], associated with the Luna16 challenge [18], which contains the annotations of 1186 nodules by four radiologists.

To the best of our knowledge, DL has not yet been applied to the problem of lung tumor detection in micro-CT data of the mouse. Furthermore, no large, publicly available murine datasets for mouse lung nodule detection exist. Nevertheless, the development of DL lung nodule detection in mice will greatly benefit ongoing co-clinical trials. To this end, we propose a DL method that uses both simulated and real data to automatically detect lung tumors in mice [19].

## 2. Materials and Methods

We first describe the animal model used in our preclinical trial along with the imaging protocol employed for micro-CT lung imaging (Section 2.1 and Section 2.2). Next, we address the data processing techniques used, including automatic lung segmentation and the generation of simulated lung tumors for training a DL model (Section 2.3 and Section 2.4). Finally, the network structure and training are described along with post processing techniques and metrics for tumor detection (Section 2.5 and Section 2.6).

### 2.1. Animal Model and Datasets

For this study, we used a collection of control mice (*n* = 10 C57BL/6) and mice with primary lung tumors (*n* = 26). The primary lung tumors were generated using intranasal administration of adenovirus expressing Cre-recombinase (Adeno-Cre, Gene Transfer Vector Core, University of Iowa) into *Kras^LSL-G12D/+^*; *p53^fl/fl^* mice. Cre-mediated recombination activates oncogenic *Kras^G12D^* by deleting LSL cassette in front of mutant *Kras* allele and deletes both copies of *Trp53* in lung epithelial cells, as described previously [20,21]. Some of the mice with lung tumors (*n* = 14) received intravenous nanoparticle-based iodinated contrast injections with a dose of 0.3 mL/25 g mouse [22,23]. Mice bearing lung tumors were imaged with micro-CT starting at 12 weeks post-intranasal adenovirus administration. All animal handling and imaging procedures were performed in accordance with protocols approved by the Duke Institutional Animal Care and Use Committee (IACUC) A173-20-08.

### 2.2. Micro-CT Imaging

Mice were scanned while breathing freely under anesthesia using 2%–3% isoflurane delivered via a nose cone. A pneumatic pillow was positioned on the animal’s thorax and connected to a pressure transducer to monitor respiratory motion and inform prospective gating. The micro-CT system and controllers were developed in-house and have been described previously [5]. The X-ray source was set to operate at 80 kVp with 40 mA current for 10 ms exposures. Projections were gated using signals from the pneumatic pillow to maximize image quality. Reconstruction was performed using Feldkamp’s (FDK) algorithm [24] using 360 projections over 360 degrees of rotation with an isotropic voxel size of 63 microns. Post-reconstruction the image volumes were processed with bilateral filtration [25] to reduce noise. The micro-CT images were converted to Hounsfield units (HU) and saved in DICOM format for processing. The Hounsfield unit is a relative quantitative measurement of X-ray attenuation used in the interpretation of CT images. The attenuation coefficient within an image voxel, X, is normalized to water and air measurements, $\mu_{water}$ and $\mu_{air}$ respectively, using Equation (1):

(1) $$X_{HU}=1000\left(\frac{X-\mu_{air}}{\mu_{water}-\mu_{air/}}-1\right)$$

The radiation dose associated with a micro-CT scan was 17 mGy per scan, i.e., a very low dose which enables longitudinal micro-CT monitoring for lung nodules.

### 2.3. Lung Segmentation

**Figure 1:**
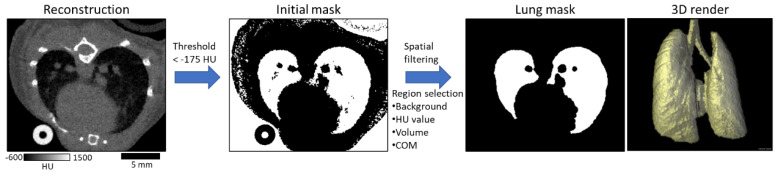
Stages of lung mask creation. The original image is thresholded to separate dense tissues from air and lungs. The resulting initial mask image is then morphologically filtered and continuous regions are sorted to identify the lung mask.

The first processing step for working with the micro-CT scans is segmentation of the lungs from the surrounding tissue. Lung segmentation is used to frame the creation of synthetic sets and to provide boundaries for detection during post-processing. We established an automated method for this task, increasing throughput and consistency. An outline of this process is described in Figure 1. The density of lung is much lower than of surrounding tissues, resulting in markedly lower grayscale values. Our CT images are presented in HU. Values for common materials include air at −1000 HU, fat at around −100 HU, water at 0 HU, soft tissues between 100 and 300 HU, and bone with values of 300+ HU. Using HU values allows for consistent processing using fixed values, and a first pass at generating a lung mask can be taken by thresholding the image by −175 HU to remove bone and soft tissue. The resulting binary mask was treated with morphological dilation and erosion to make the mask internally continuous, allowing vasculature and nodules to be included in the map, and to remove features too small to be considered lungs. Next, each of the remaining continuous regions was analyzed to determine which contained the lungs. Due to the small size of a mouse, much of the body appears in the micro-CT image, making detection of the lungs a non-trivial part of the task to create a lung mask. To minimize the chance of selecting incorrect regions as the lung mask, the continuous regions in the thresholded image were rejected if they did not meet the following three criteria: (1) No region can be connected to the background air around the mouse. This serves to reject many of the air-filled portions of the cradle holding the mouse. (2) The mean HU value within a region must be within a range of −175 to −600 HU. This filters out regions which contain only air since they would have values much lower than this. (3) The volume of a region must be within 200 to 1200 mm^3^. These values were chosen empirically based on the range of lung volumes seen in previous work with mice [5]. Additionally, if more than two continuous regions remained after the previous three criteria, further filtering was performed using the spatial distribution of the coordinates for each region. Each region was clustered into two groups of similar size using k-means. It is expected that each group would constitute a lung (left and right) and the distance between the center of each group was used to weight selection of regions with two distinct centers of mass.

### 2.4. Data Augmentation with Simulated Image Generation

Machine learning is a powerful tool for complex tasks such as lung nodule detection. However, it is often difficult to obtain a sufficiently large and varied amount of labeled data for robust training. Large databases of clinical CT are available for analysis of lung tumors [17]; however, no such repositories currently exist for preclinical data. Instead, our approach was limited to working with data collected on our own system. To augment this data, we adopted a strategy which employs simulated data. Lung tumor detection can be studied using simulated tumors inserted into temporally resolved micro-CT datasets of healthy lungs. A similar procedure has been described in detail by us in [5], and comparable procedures have been used to create databases of lung nodules for machine learning applications in clinical CT [26].

Scans of mice bearing lung tumors were analyzed and labeled for training. A researcher experienced in murine lung imaging labeled tumor locations using the ITK-Snap segmentation software (www.itksnap.org (accessed on 12 March 2020)) [27]. Due to the small sizes of the tumors, delineating exact tumor margins was difficult; therefore, general tumor locations were marked for detection rather than for segmentation. Label maps were saved as binary image volumes, which preserved tumor location, relative size, and shape.

**Figure 2:**
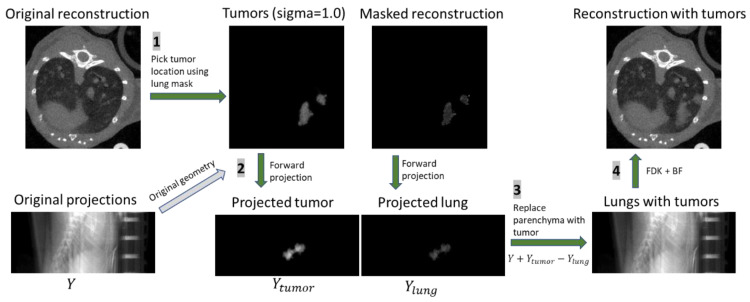
Diagram showing the generation of simulated lung tumors. (1) A scan of a healthy mouse is used as the base. (2) Tumors are added to empty volumes which are projected into the same space as the original projections. (3) The tumor is added and the lung subtracted from the original projections. (4) The reconstruction with lung tumors is performed via FDK followed by bilateral filtration (BF).

Using our micro-CT system, scans of six healthy mice were taken from previously acquired sets [5] in which to place tumors. These mice were imaged without the addition of contrast. From the same study, a bank of seven manual segmentations of real lung tumors was created for incorporation into the healthy reconstructions. The method for inserting tumors into healthy lung scans is outlined in Figure 2. To augment this small bank of tumors, they were first subjected to transformations involving random rotations, skews, and scaling, resulting in tumors with a range of sizes (0.03 to 70 mm^3^, with a median size of 0.65 mm^3^ and 90% of tumors less than 10 mm^3^). Transforming the tumors altered their native gray values and textures. To address this, the tumors were retextured with Gaussian noise and Gaussian smoothing was applied to the tumor masks to make the augmented tumors statistically similar to the originals. This step also served to blend the original image and tumor together, alleviating abrupt differences in gray values. The augmented tumors were randomly placed within the lung mask of a randomly selected healthy lung scan (Figure 2, part 1). The permitted locations for the tumor were such that it could not be placed more than 10% outside the lung-mask or overlapping with other tumors. In mice, lung tumors are often located along the boundaries of the lungs, and near other tumors. To simulate this property, choices of tumor location were weighed using the inverse Euclidean distance to the nearest lung mask boundary. Positions nearest an edge of the mask were selected with higher frequency than those far from a mask boundary. To increase the likelihood of creating groups of tumors, as is often seen with lung nodules, previously placed tumor locations were removed from the lung mask, creating new boundaries with high probability for additional tumors.

**Figure 3:**
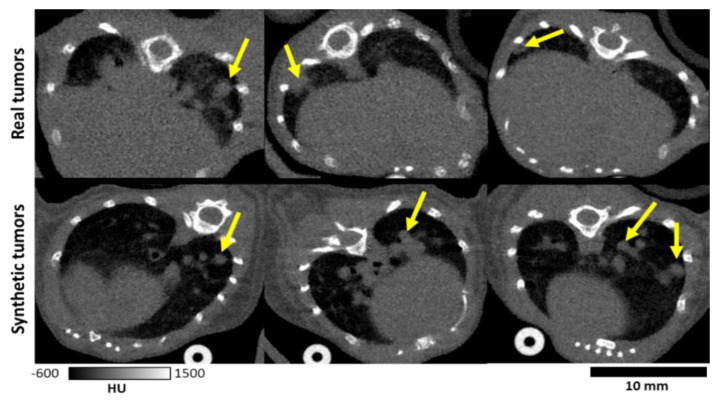
Comparison of real and simulated tumors. Tumors are marked with yellow arrows. The simulated tumors are shown to contain similar sizes and textures as the real tumors.

After all lung nodules were positioned using the lung mask, a 3D volume containing only the tumors was forward projected for all angles in a cone-beam geometry identical to that of the original micro-CT acquisition of the healthy mouse. Similarly, a second volume which contained the parenchyma behind the synthesized tumors was created and forward projected (Figure 2, part 2). The original projections were altered by adding the tumor projections, while the tissue which would be covered by the tumors was subtracted (Figure 2, part 3). Reconstruction via filtered backprojection of the resulting projections followed by bilateral filtration yielded simulated tumor images (Figure 2, part 4). After, additional augmentation was performed by applying a random magnification to the reconstructed volume of ±10%. The final image was cropped around the lung volume with a padding of 30 voxels on each size. The binary maps of the simulated lung tumors were used as labels for DL training and served as the ground truth for assessing results of detection. Figure 3 compares examples of mice with real and simulated lung tumors and shows that our simulated sets are realistic. We created a total of 60 simulated training sets of which 13 were set aside for training validation. Each set contained two simulated tumors.

### 2.5. Network Structure

**Figure 4:**
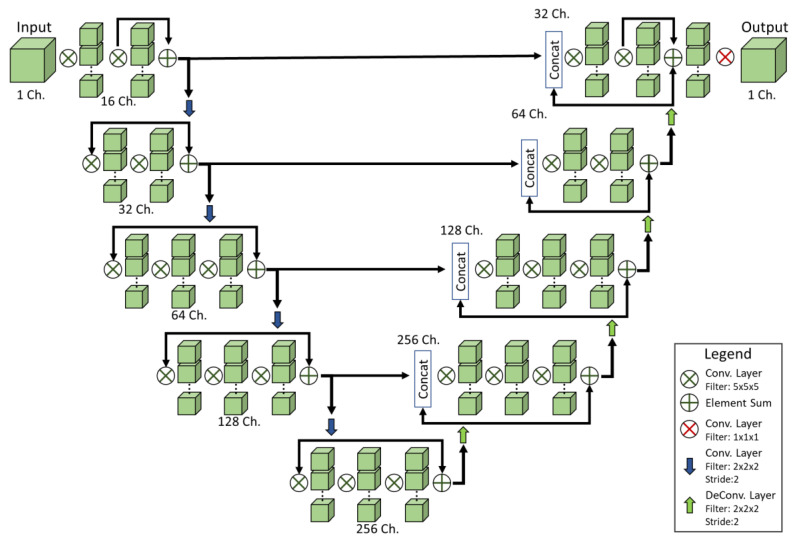
Network structure. The V-Net consists of an encoder/decoder configuration connected by skip connections. Volumetric image patches are processed with 3D convolution kernels. The number of feature maps (channels) at each stage increases with the depth of the network. Convolution layers consist of convolution followed by batch normalization and activation via PReLU. The output of the network is a probability map with floating point values [0,1] and the same dimensions as the input.

With the goal of providing automated lung tumor detection in mice we implemented a CNN and conducted supervised training using both real and simulated datasets. Here we present our CNN structure and provide information on training and testing of a small number of DL models. The network architecture we elected to use in this work has been previously published as the V-Net CNN, which has seen success in detecting lung tumors in clinical data [28]. The V-Net, shown in Figure 4, is comprised of an encoder/decoder structure. The convolution operations in this network have volumetric kernels with fixed size (5 × 5 × 5). In the encoder, convolution operations extract image information and reduce the size of the data. The decoder then decompresses that data back to its original size. The network can be divided into stages, with each stage representing a distinct image resolution. A stage is composed of a block of one to three convolution layers. Residual connections are placed around every stage, adding the input feature maps to the output of each block of convolution layers. Residual connections have been found to address problems with vanishing gradients and greatly increase the speed at which networks can be trained [29]. Data are reintroduced into the decoder at each stage via skip connections from the encoder. The network has five stages, each representing the data at a different resolution via downsampling and upsampling. In the encoder, downsampling operations are performed via convolution with 2 × 2 × 2 kernels applied with a stride of 2. Upsampling in the second half of the network is performed with deconvolution layers with 2 × 2 × 2 kernels and a stride of 2. Batch normalization was performed after each convolution layer to center the data [30]. Nonlinearities were introduced to the signal after each layer via parametric rectified linear activation units, or PReLUs. PReLUs are similar to leaky ReLUs in that they improve gradient propagation; however, the leakage of negative values is controlled by a learned parameter [31]. The network terminates with convolution using a 1 × 1 × 1 kernel, which reduces the numbers of feature maps to a single output channel.

The network processes volumetric patches measuring 96 × 96 × 96 voxels. On average, training volumes contained few tumors; therefore, random patches were selected such that they contain all or part of a tumor in 80% of cases. This type of selection serves as a form of augmentation and incentivizes the network to be more aggressive with its predictions. Additional augmentation was performed during training via random image flips in the coronal plane. Because the input images were already in a standard HU scale (see Equation (1)), standard zero-mean and unit-variance normalization was not required. Instead, normalization was performed by simply dividing the inputs by 1000 to bring their magnitude into a range closer to the desired network output. To increase the size of the receptive field and reduce the effects of noise, input images were resampled from 63 µm to 100 µm isotropic voxels. This was carried out with little loss in resolution, as this value is still beneath the system’s Modulation Transfer Function (MTF) at 10% [4]. The network output is an image block of the same size showing the tumor segmentation. Training labels were the corresponding binary maps indicating the location of lung nodules.

Due to the small size of available real data (36 scans, of which 26 present tumors), small variations in the test set could produce widely different results in terms of quality metrics. For this reason, a single test set will poorly indicate how well a network will generalize to data outside of its training and test sets. To combat this, we have trained our networks using a K-fold cross validation strategy. The data were split into training (80%) and test (20%) sets for which a network was trained and evaluated. This process was performed five times, with different training and test splits. In this way every scan was part of the test set for one of the networks. The performance of all K-fold networks on their respective test sets were aggregated to give better significance to the results.

Networks were trained and evaluated in four competing ways. (1) The network was trained using only simulated data. This network was trained only once using the full simulated dataset and was evaluated on all the real data. This method gives a direct measure of the utility of the simulated datasets. This network was evaluated as though it were a k-means network, using five test groups for consistency with the following networks. (2) For comparison, a set of K-fold networks were trained using only real data. (3) Another set of K-fold networks were trained using combined simulated and real datasets. The real data was split using K-folds while the entire simulated training set was added to the training data for each network. Evaluation was performed using real data. (4) Finally, the network trained only on simulated data was retrained on real data in a K-fold transfer learning step. Training networks in this variety of ways allows the utility of the simulated data to be assessed.

The network had a total of 4.4 million trainable parameters. This implementation of V-Net was coded using Tensorflow [32]. The networks were trained for 5000 epochs, with the test set being monitored to avoid overfitting. We employed a Dice loss function to train the network. Dice measures the overlap between label T and predicted P binary maps and is given by Equation (2):

(2) $$D=\frac{2\left|P\cap^{}T\right|}{\left|P\right|+\left|T\right|}$$

where |P| and |T| represent the cardinality of the predicted and label volumes, respectively. The network weights were updated using the Adam optimizer with an initial learning rate of 0.01 and momentum of 0.9 with a batch size of 3. The learning rate was reduced by 10% after each 100 epochs. To prevent overfitting, dropout was employed at 1% after each activation function. The network was trained on a stand-alone workstation equipped with four NVIDIA Titan Xp graphics cards. Networks were trained in parallel, using one GPU per network. It took 15 h to train each network.

Once trained, evaluating the network required breaking each test volume into multiple overlapping patches to pass through the network. Patches were extracted with a stride of 12 in each direction, resulting in 100+ patches per volume. Once processed, patches were organized back into a single volume and overlapping regions were averaged. In this way the model can evaluate 1.6 patches/second, taking about 1 min to process a 300 × 300 × 300 lung volume.

### 2.6. Detection Post Processing

The prediction maps required some post-processing to achieve best results. The first step rejects all detections outside of the calculated lung mask. Next, a decision threshold is applied to convert the output of the network from a floating-point probability for each pixel from [0,1] to binary detection maps. To reduce the number of false positives, any continuous regions with a volume less than 0.15 mm^3^ are rejected since nodules of that size cannot be reliably imaged on our micro-CT system [4]. In this work detection is constituted as a direct or a near hit. A direct hit occurs when there is an overlap between labeled tumors and detection maps, whereas a near hit occurs when the detected nodule lies close to the labeled nodule. This is defined as being within 1.5 times the radius of the center of the labeled tumor [11,14]. Predicted regions outside of this space are considered false positives.

We evaluated the performance of our model for lung tumor detection using precision and recall curves, as well as receiver operating curves (ROC). Precision and recall, sometimes called positive predictive value and sensitivity, were calculated from the numbers of true positives (TP), false positives (FP), and false negatives (FN). The equations for precision and recall are shown in Equations (3) and (4):

(3) $$precision=\frac{TP}{TP+FP}$$

(4) $$recall=\frac{TP}{TP+FN}$$

It is necessary to maximize the performance for the final detection task. This is carried out by selecting an optimal decision threshold. A threshold was calculated for each K-fold network to allow for the processing of each network’s test set. These metrics are found by processing each network’s training data, sweeping detection thresholds on the outputs, and performing the detection calculation for each. Precision and recall can be plotted as a function of decision threshold. The threshold at the intersection of precision and recall gives the best combined performance of the two metrics and most agreement between the predicted segmentation and label images. This threshold gives the highest true positive and lowest false positive performance, maximizing the quality of the detection.

## 3. Results

**Figure 5:**
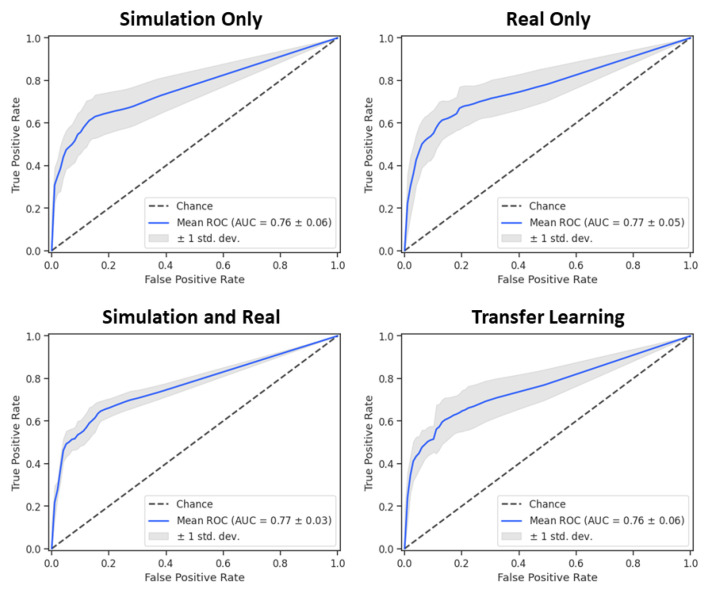
Receiver operating curves for networks trained using synthetic data, real data, combinations of the two, and transfer learning. The mean and standard deviation for the five K-fold networks are shown in blue and gray, respectively. All four training cases are shown to be very similar.

Detection performance of real lung tumors as given by the ROCs is shown in Figure 5. Curves are shown for each of the four training cases: training on simulated data, real data, combinations of the two, and transfer learning. In each case the performance of the five K-fold networks is shown using the mean and standard deviation. The AUC, which provides an aggregate score of network performance, is similar in each case and within the standard deviation of the measurements over the K-folds. The greatest consistency (lowest standard deviation) is shown from networks trained on both real and simulated data. These results indicate that simulated data provide similar quality networks as the real data, and that additional training data lead to more consistent results.

**Figure 6:**
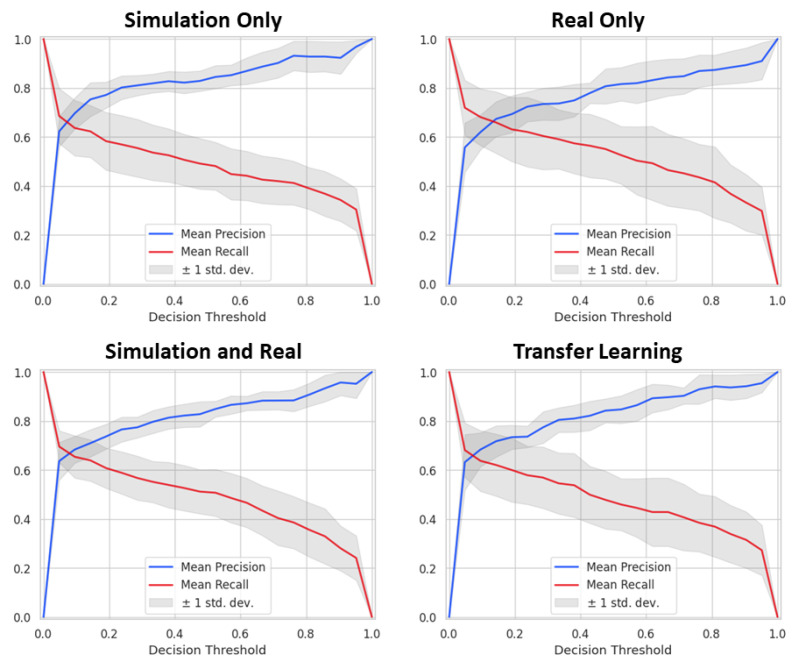
Precision and recall plotted over decision threshold. Solid lines represent the mean performance of the five K-fold networks, while the gray areas around them show the standard deviation. The chosen decision threshold for every network fell between 0.05 and 0.1.

Precision and recall are shown in Figure 6 to demonstrate changes in performance with decision threshold. Plots are presented for all four training strategies. The intersection of precision and recall denotes an optimal decision threshold which maximizes both metrics along with combined metrics, such as Dice score. This crossing point threshold was used for lung tumor detection. The threshold for the network trained only on simulated data was calculated using real data, which gave much better performance than using simulated data. Note that the precision-recall curves have similar values, and their intersection, which defines an optimal threshold for detection, is almost identical, indicating similar performance between the training cases.

**Figure 7:**
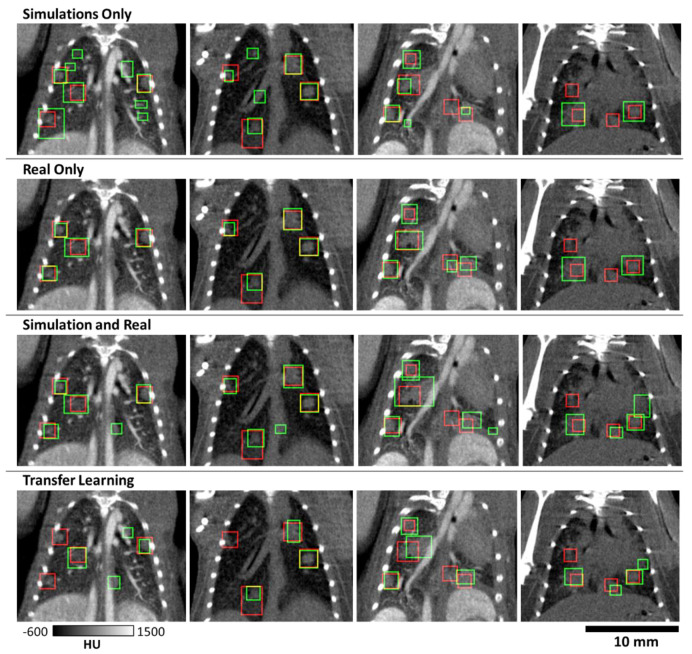
Samples showing bounding boxes for hand-curated labels (red) and predicted tumors (green) marked by bounding boxes. Four mice are shown as columns, showing performance with and without the addition of contrast. Though there is variation in network predictions, detection performance is largely similar between training cases. A major source of error is the similarity of vasculature and nodules in these images.

Examples of tumor detection in test mice with primary lung tumors are shown in Figure 7. These results are shown for four animals scanned with and without contrast. The decision threshold for these images was found via the intersection of precision and recall for each network (Figure 6). Note that the trained V-Net is successful in detecting the majority of lung tumors. The dimension of the predicted bounding box did not always match the label, but there was sufficient overlap to constitute detection. The results on this data show that there are some larger tumors that are detected as two separate tumors by the CNN, which did not affect the performance of the detection task.

Using the decision threshold calculated via the precision and recall curves, all real data were run through their related K-fold networks and detections were calculated. This resulted in the whole dataset being processed as test data. The aggregate detection performance for each training strategy is summarized in Table 1. This table lists the number of true positives, false positives, and false negatives for the entire real dataset. These values are used to calculate precision, recall, and Dice scores using equations 2–4 for each training strategy. These data show that using only simulations provides the most similar precision and recall metrics, with an overall performance which is slightly better than using real data alone (Dice of 0.643 vs 0.623). Training using only real data results in a large number of false positives compared to other strategies, negatively impacting its precision. Using both simulated and real data, with joint training or transfer learning, provides the best performance according to the Dice scores (0.661 and 0.665, respectively). Additionally, precision on average increased 8% against networks trained on one type of data. This indicates that the increased number of training sets improves performance. Transfer learning has the highest overall Dice score; however, higher sensitivity as seen with joint training may be preferable for detection tasks so that nodules are not missed.

**Figure 8:**
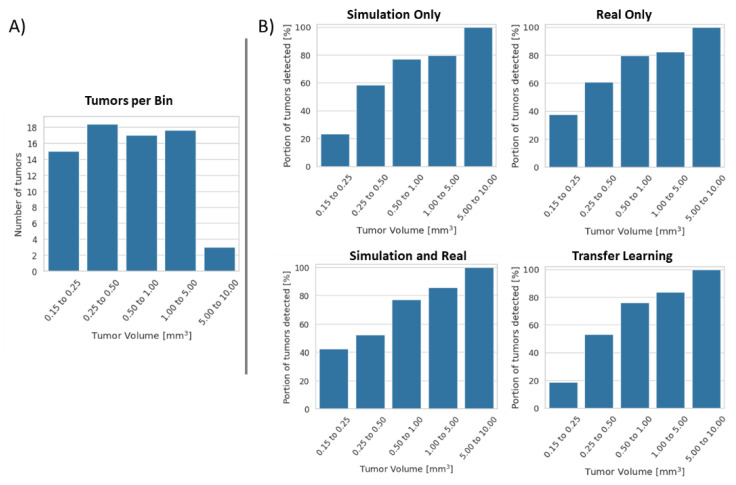
Distribution of tumor sizes and detection rates in experimental data. (**A**) Most tumors fell in the range of 0.15 to 1 mm^3^; however, several larger tumors did exist in the data. (**B**) Percent of tumors detected by networks trained using each strategy. False positives are not represented in these measurements. The network trained on simulated data struggled to find the smallest tumors, as did the network trained via transfer learning.

An important aspect of lung nodule detection is how the size of the nodule affects detection. Figure 8A shows the distribution of tumor sizes in the real dataset. The majority of the tumors are small and are measured at less than 1 mm^3^; however, a few tumors exist with sizes up to 10 mm^3^. Figure 8B shows an analysis of detection efficacy per tumor size. We have divided our tumors in five bins depending on their volumes. The biggest difference in performance is seen for the smallest tumors. As expected, detection is worse for smaller tumors overall; however, networks trained with real data are much better at this task. In the portion of tumors smaller than 0.25 mm^3^, 23% vs. 38% were detected for networks trained on simulation vs. real data. The network trained on both real and simulated data performed best for detection of small tumors, with 41% detected. Oddly, the worst small tumor performance is seen from the transfer learning networks, with just 19% detection.

## 4. Discussion

Our work suggests that DL can offer a promising approach for fast and relatively accurate detection of lung tumors in mice. These results have been validated by training networks with four strategies involving simulated data, real data, a combination of the two, and pre-training a network on simulated data followed by refinement with real data. We have shown that our method of creating virtual nodules in realistic simulations can provide an excellent way to improve network performance when there is a scarcity of data. A network trained on simulated data alone can achieve performance near that of using only real data, with an AUC of 0.76 vs. 0.77 for real data (Figure 5). Additionally, supplementing real training data with simulated data, either in a single training step or in two via transfer learning, can lead to improved performance over networks employing either of those data types alone. We note that our simulation-based training was quite effective, providing training performance that was close to all the training cases. This indicates that our virtual nodules matched the characteristics and distribution of lung nodules in our real data. The networks were trained using a Dice loss function, which is often employed for segmentation tasks [33]. We opted to use the network for detection. We note that in many cases detection is the preferred clinical solution for lung nodules and is frequently studied for computer-aided diagnosis [34,35].

We found that the detection performance was linked to the size of the nodule. Detection of small tumors is a more difficult task than for large tumors, with 19% to 38% of small tumors being detected. This performance jumps to over 80% for tumors measuring 1 to 5 mm^3^, with all tumors larger than 5 mm^3^ being detected. For longitudinal scanning, this property can be used to retrospectively identify growing tumors which were too small for detection in previous scans.

We note, however, that our detection metrics are not on par with those seen in clinical CT. For example, one clinical study reported recall values of 0.95 [16] vs. the 0.71 value we have reported for a network trained with both real and simulated data (Table 1). The discrepancy between metrics for clinical and preclinical work is attributed to challenges associated with murine lung imaging. These challenges include the small size of the animal and nodules being scanned. Without unreasonable increases in radiation dose, reconstructions will be inherently much noisier than for clinical CT, confounding analysis. Additionally, while respiratory gating is employed, the longer scan times (~5 min) for preclinical imaging mean that motion blurring is of greater concern than in clinical scans completed in seconds. As a confounding factor, vasculature in mice is of a similar scale and density as nodules (Figure 3), whereas tumors may be much larger in humans. Due to these properties, vasculature may be easily mistaken for nodules, and vice versa as is shown in Figure 7. These properties can confound both network performance and make manual segmentation of tumors for labels challenging, which in turn hurts numerical performance.

## 5. Conclusions

Improved accuracy of our deep learning model may be achieved by using a larger training set with real lung tumors. Moreover, including in simulations some cases with thickened bronchioles and vascular shadows may be a way to increase the discrimination performance of our DL approach in separating nodules from other lung pathologies. We will next apply our tools in future studies as a computer-aided diagnostic tool and use future results to refine training. This will increase the efficacy of our methods over time. Such DL approaches to automatic detection and segmentation will speed up quantitative analysis in the preclinical arm of co-clinical trials while reducing potential human bias. These types of DL approaches could also be extended to, for example, interstitial lung diseases including asthma, idiopathic pulmonary fibrosis (IPF) and chronic obstructive pulmonary disease (COPD) mouse models.

## Figures and Tables

**Table 1:** Detection performance for networks trained using simulations, real data, a combination of both, and transfer learning. Decision thresholds were used as found from the training data for each network.

Training	True Positives	False Positives	False Negatives	Precision	Recall	Dice
**Simulation Only**	138	84	69	0.622	0.667	0.643
**Real Only**	148	120	59	0.552	0.715	0.623
**Simulation and Real**	147	91	60	0.618	0.710	0.661
**Transfer Learning**	139	72	68	0.659	0.671	0.665

## Data Availability

The data presented in this work are available by request at https://civmvoxport.vm.duke.edu, and the code used for training and analysis can be found at https://github.com/mdholbrook/lung-tumor-detection.
